
JOURNAL INFORMATION
==============================
NLM Title Abbreviation: CPT Pharmacometrics Syst Pharmacol
Journal ID: CPT Pharmacometrics Syst Pharmacol
NLM Journal ID: 101580011
Journal ID: PSP4

CPT: Pharmacometrics & Systems Pharmacology

EISSN: 2163-8306
Publisher: Wiley

ARTICLE INFORMATION
==============================
PMCID: PMC11474803
DOI: 10.1002/psp4.12985
Article ID: PSP412985
Article version: 1
Subjects: Issue Information, Issue Information

Issue Information

Electronic publication date: 2024 Feb 13
Publication date: 2024 Feb
Volume: 13
Issue: 2
First page: 183
Last page: 186
Copyright: © 2024 ASCPT
License: This is an open access article under the terms of the http://creativecommons.org/licenses/by/4.0/ License, which permits use, distribution and reproduction in any medium, provided the original work is properly cited.
License URL: https://creativecommons.org/licenses/by/4.0/

Figure count: 0
Table count: 0
Page count: 3

source-schema-version-number: 2.0
cover-date: February 2024
details-of-publishers-convertor: Converter:WILEY_ML3GV2_TO_JATSPMC version:6.4.9 mode:remove_FC converted:15.10.2024

==============================

An Official Journal of ASCPT and ISoP

### CPT: Pharmacometrics & Systems Pharmacology

Open Access

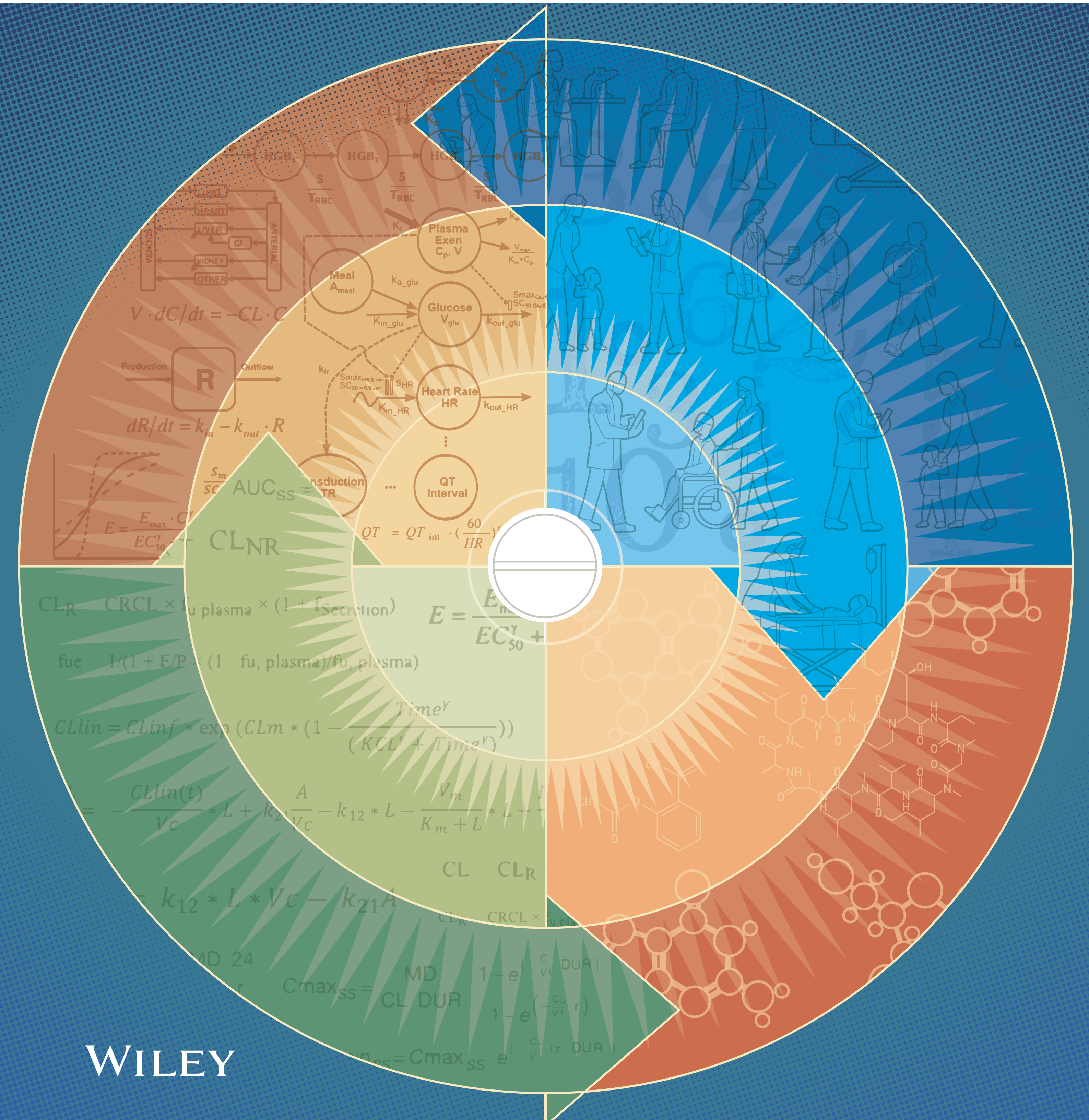

WILEY

### CPT: Pharmacometrics & Systems Pharmacology

WILEY

### Editor-in-Chief

France Mentré, MD, PhD, *School of Medicine of University Paris Diderot, Paris, France*

### Deputy Editor-in-Chief

Karen Rowland Yeo, PhD, *Certara UK Ltd., Sheffield, UK*

### Associate Editors

Paolo Denti, PhD, *University of Cape Town, Cape Town, South Africa*
Rajat Desikan, PhD, *GSK, Stevenage, UK*
Jonathan French, ScD, *Johnson & Johnson Innovative Medicine, Spring House, PA, USA*
Stefanie Hennig, MSc, PhD, *Certara Strategic Consulting, Brisbane, Australia*
Priya Jayachandran, PharmD, *Regeneron Pharmaceuticals, Inc., San Francisco, CA, USA*
Andrew Hooker, PhD, *Uppsala University, Uppsala, Sweden*
Sriram Krishnaswami, PhD, *Pfizer, Groton, CT, USA*
Ping Zhao, PhD, *Bill & Melinda Gates Foundation, Seattle, WA, USA*

### Editors-in-Training

Brian Cicali, PhD, *University of Florida, College of Pharmacy, Orlando, FL, USA*
Jane Knöchel, PhD, *AstraZeneca , Mölndal, Sweden*

### American Society for Clinical Pharmacology and Therapeutics

***Chief Executive Officer***
Abigail W. Gorman, *Alexandria, VA*

***Chief Operating Officer***
Elise S. Laffman-Johnson, *Alexandria, VA*

***Managing Editor***
Alethea B. Gerding, *Alexandria, VA*

***Deputy Managing Editor***
Alaina Webster, *Alexandria, VA*

***Senior Editorial Coordinator***
Aimee Greene, *Alexandria, VA*

### Editorial Board

Vivaswath S. Ayyar, PhD, *Janssen Research & Development, Spring House, PA*
Robert Bies, PharmD, PhD, *State University of New York at Buffalo, Buffalo, NY, USA*
Marc Birtwistle, PhD, *Clemson University Clemson, SC, USA*
Yuan Chen, PhD, *Genentech South San Francisco, CA, USA*
Marylore Chenel, PharmD, PhD, *Pharmetheus Uppsala, Sweden*
Emmanuel Chigutsa, PhD, *Eli Lilly and Company, Indianapolis, IN, USA*
Teresa Dalla Costa, PhD, *Federal University of Rio Grande do Sul - UFRGS Porto Alegre, Brazil*
Narendra M Dixit, PhD, *Indian Institute of Science, Bengaluru, India*
Anne-Gaelle Dosne, PharmD, PhD, *Janssen Research & Development, Beerse, Belgium*
Thomas Dumortier, Eng, MSc, *Novartis Pharma AG, Basel, Switzerland*
James M. Gallo, PharmD, PhD, *University at Buffalo, Buffalo, NY, USA*
Wei Gao, PhD, *EMD Serono, Billerica, MA, USA*
Jérémie Guedj, PhD, *INSERM, Paris, France*
Manish Gupta, PhD, FCP, *GenMab; Princeton, NJ, USA*
Bart Hendriks, PhD, *Novartis Institutes for BioMedical Research Cambridge, MA, USA*
Nick Holford, MBChB, *The University of Auckland Auckland, New Zealand*
Brett A. Howell, PhD, *DILIsym Services Division, Simulations Plus, Inc., Research Triangle Park, NC, USA*
Manuel Ibarra, PhD, *Universidad de la República, Montevideo, Uruguay*
Siv Jönsson, PhD, *Uppsala University Sweden, Uppsala, Sweden*
Kristin Karlsson, PhD, *Uppsala University Uppsala, Sweden*
Helen Kastrissios, PhD, *QuanTx Consulting, Brisbane, Australia*
Ron Keizer, PhD, *InsightRX San Francisco, CA, USA*
Steven E. Kern, PhD, *Global Health Labs, Bellevue, WA, USA*
Akash Khandelwal PhD, *UCB Biosciences GmbH, Monheim am Rhein, Germany*
Andrzej Kierzek, PhD, *Certara QSP; Sheffield, United Kingdom*
Qi Liu, PhD, *US Food and Drug Administration, Silver Spring, MD*
James Lu, PhD, *Genentech, Inc., South San Francisco, CA, USA*
Rajanikanth (Raj) Madabushi, PhD, *US Food and Drug Administration, Silver Spring, MD*
Efthymios Manolis, MSc, *European Medicines Agency London, United Kingdom*
Samer Mouksassi, PharmD, PhD, *Certara, Cairo, Egypt*
Jackson K. Mukonzo, PhD, *Makerere University, Kampala Uganda*
Joakim Nyberg, PhD, *Pharmetheus, Uppsala, Sweden*
Elsje Pienaar, PhD, *Weldon School of Biomedical Engineering, Purdue University, West Lafayette, IN, USA*
Daniel Scholes Rosenbloom, PhD, *Merck & Co., Rahway, NJ, USA*
Joe Standing, PhD, *University College London London, United Kingdom*
Natasha Strydom, PhD, *Amgen, Inc., San Francisco, CA, USA*
Yuichi Sugiyama, PhD, *Riken Innovation Center Sugiyama Laboratory Yokohama, Japan*
Elin Svensson, PhD, *Uppsala University, Uppsala, Sweden*
Joel Tarning, PhD, *Mahidol-Oxford Tropical Medicine Research Unit Bangkok, Thailand*
Nadia Terranova, PhD, *Merck KGaA, Lausanne, Switzerland*
Michael Tortorici, PharmD, PhD, *Aro Biotherapeutics, Philadelphia, PA, USA*
Coen van Hasselt, PhD, *Leiden University, Leiden, the Netherlands*
Paolo Vicini, PhD, MBA, *Confo Therapeutics, Ghent, Belgium*
Camille Vong, PhD, *Novartis AG Pharma, Basel, Switzerland*
Xinyuan (Susie) Zhang, PhD, *Daiichi Sankyo, Inc. Basking Ridge, NJ, USA*

### Overview

*CPT: Pharmacometrics & Systems Pharmacology* (PSP) is an official open access journal of the American Society for Clinical Pharmacology and Therapeutics (ASCPT) and International Society of Pharmacometrics (ISoP).

*CPT: Pharmacometrics & Systems Pharmacology* is a cross-disciplinary journal devoted to publishing advances in quantitative (e.g., modeling and simulation) methods as applied in pharmacology, physiology and therapeutics in humans. The journal welcomes original research articles, reviews and tutorials that bridge the following areas: pharmacometrics, modeling and simulation as applied to the design and evaluation of clinical trials, systems pharmacology modeling, particularly with a mechanistic link to human (patho)physiology, disease modeling, "population" or mixed-effects pharmacokinetics and pharmacodynamics (PKPD) modeling, modeling and simulation to support translational research, physiologically-based pharmacokinetics (PBPK), model-based meta-analyses of clinical trials, mechanism-based pharmacokinetic-pharmacodynamic modeling, computational pharmacology, bioinformatics, comparative efficacy, effectiveness and cost-effectiveness. Systems pharmacology may involve the application of systems biology approaches to study drug activities, targets and effects. The discipline is often defined with reference to engineering and pharmacological principles as the quantitative analysis of the dynamic interactions between drugs and a biologic system that aims to understand the behavior of the system as a whole. The common focus will be on quantitative methods that improve our understanding of pharmacology and therapeutics in humans.

**Abstracting and Indexing**
- Embase (Elsevier)
- SCOPUS (Elsevier)
- MEDLINE/PubMed indexing

**Society Information**

The American Society for Clinical Pharmacology and Therapeutics (ASCPT) was founded in 1900, and consists of over 2,200 professionals whose primary interest is to advance the science and practice of clinical pharmacology and translational medicine for the therapeutic benefit of patients and society. ASCPT is the largest scientific and professional organization serving the disciplines of Clinical Pharmacology and Translational Medicine.

**All articles published in this journal are freely available to read, download, and share. Articles are published under the terms of the Creative Commons Attribution Non-Commercial License which permits use, distribution, and reproduction in any medium, provided the original work is properly cited and is not used for commercial purposes.**

**Disclaimer**

The Publisher, the American Society for Clinical Pharmacology and Therapeutics, the International Society of Pharmacometrics, and Editors cannot be held responsible for any errors in or any consequences arising from the use of information contained in this journal. The views and opinions expressed do not necessarily reflect those of the Publisher, the American Society for Clinical Pharmacology and Therapeutics, the International Society of Pharmacometrics or Editors, neither does the publication of advertisements constitute any endorsement by the Publisher, the American Society for Clinical Pharmacology and Therapeutics, the International Society of Pharmacometrics, Editors, or Authors of the products advertised.

**Publisher**

*CPT: Pharmacometrics & Systems Pharmacology* is published by Wiley Periodicals, LLC, 101 Station Landing, Suite 300, Medford, MA 02155; Telephone: 781-388-8200; Fax: 781-388-8210.

CONTENTS

CPT: Pharmacometrics & Systems Pharmacology

### PERSPECTIVE

### PERSPECTIVE

**187** Confounded exposure metrics
**Matthew R. Wiens, Jonathan L. French and James A. Rogers**

### TUTORIAL

### TUTORIAL

**192** Bayesian estimation in NONMEM
**Curtis K. Johnston, Timothy Waterhouse, Matthew Wiens, John Mondick, Jonathan French and William R. Gillespie**

### RESEARCH

### ARTICLES

**208** Adult and pediatric physiologically-based biopharmaceutics modeling to explain lamotrigine immediate release absorption process
**Edilainy Rizzieri Caleffi-Marchesini, Amanda Antunes Herling, Julia Macente, Rodolfo Hernandes Bonan, Priscila de Freitas Lima, Rafaela Moreno, Veriano Alexandre, Nitin Bharat Charbe, Fernanda Belincanta Borghi-Pangoni, Rodrigo Cristofoletti and Andréa Diniz**

**222** Multistate modeling for survival analysis in critically ill patients treated with meropenem
**Yaru Peng, Iris K. Minichmayr, Han Liu, Feifan Xie and Lena E. Friberg**

**234** Assessment of CYP3A-mediated drug interaction via cytokine (IL-6) elevation for mosunetuzumab using physiologically-based pharmacokinetic modeling
**Yuan Chen, Fang Ma, Nicholas Jones, Rong Deng, Chunze Li and Chi-Chung Li**

**247** Use of physiologically-based pharmacokinetic modeling to understand the effect of omeprazole administration on the pharmacokinetics of oral extended-release nifedipine
**Ming-Liang Tan, Zongming Gao, Andrew Babiskin, Myong-Jin Kim, Lanyan Fang, Lei Zhang and Liang Zhao**

**257** Machine learning–based integration of network features and chemical structure of compounds for SARS-CoV-2 drug effect analysis
**Julian Späth, Rui-Sheng Wang, Maeve Humphrey, Jan Baumbach and Joseph Loscalzo**

**270** Optimizing the design of a pharmacokinetic trial to evaluate the dosing scheme of a novel tuberculosis drug in children living with or without HIV
**Grace Montepiedra, Elin M. Svensson, Weng Kee Wong and Andrew C. Hooker**

**281** Model-based meta-analysis using latent variable modeling to set benchmarks for new treatments of systemic lupus erythematosus
**Kosalaram Goteti, Ramon Garcia, William R. Gillespie, Jonathan French, Lena Klopp-Schulze, Ying Li, Cristina Vazquez Mateo, Sanjeev Roy, Oliver Guenther, Lisa Benincosa and Karthik Venkatakrishnan**

**296** Potassium homeostasis and therapeutic intervention with sodium zirconium cyclosilicate: A model-informed drug development case study
**Lindsay E. Clegg, Lulu Chu, Mats Nagard, David W. Boulton and Robert C. Penland**

**308** Computational drug discovery on human immunodeficiency virus with a customized long short-term memory variational autoencoder deep-learning architecture
**Mucahit Kutsal, Ferhat Ucar and Nida Kati**

**317** Physiologically-based pharmacokinetic model of sparsentan to evaluate drug–drug interaction potential
**Nikunjkumar K. Patel, Kuan-Fu Chen, Shang-Chiung Chen and Kai Liu**
